# The Intersection of SGLT2 Inhibitors, Cognitive Impairment, and CKD

**DOI:** 10.3389/fneur.2022.823569

**Published:** 2022-06-21

**Authors:** J. Ariana Noel, Ingrid Hougen, Manish M. Sood

**Affiliations:** ^1^Department of Nephrology, The Ottawa Hospital, Ottawa, ON, Canada; ^2^Postgraduate Medicine, University of Ottawa, Ottawa, ON, Canada

**Keywords:** chronic kidney disease, cognitive impairment, dementia, albuminuria, SGLT2 inhibitor

## Abstract

Impairment in cognition and decline in kidney function often converge in the aging individual with chronic kidney disease (CKD). Cognitive impairment (CI) may be preventable through modification of health behaviors and risk factors that contribute to the vascular disease burden. CKD patients often have multiple coexisting comorbid conditions contributing to vascular risk. These comorbidities include hypertension, diabetes, cerebrovascular disease, and cardiovascular disease. Emerging evidence suggests that the management and prevention of vascular risk factors and cardiovascular diseases may indirectly contribute to the prevention of CI in CKD. Sodium glucose transport protein 2 inhibitors (SGLT2i) are emerging as the standard of care for selected individuals with CKD, type 2 diabetes (T2DM), and heart failure with rapidly expanding indications being actively investigated. In this narrative review, we examine the intriguing hypothesis that SGLT2i demonstrate potential disease modifying properties in CI among individuals with CKD.

## Introduction

Cognitive impairment (CI) is the loss of brain functions including concentration, attention, executive function, verbal fluency, and memory beyond what is expected for age (1–3). Chronic kidney disease (CKD) is defined by two sequential measures of estimated glomerular filtration rate (eGFR) <60 ml/min/1.73 m^2^ or albuminuria >30 mg/g more than 90 days apart (4, 5). CKD is a worldwide emerging epidemic with an estimated incidence between 8 and 16% of the population with a mortality rate of 42% (2, 5). CKD is not only related to increased mortality, but to other comorbidities associated with CI such as T2DM, hypertension, stroke, and heart failure (6–8). End stage kidney disease (ESKD) patients have significantly worse memory performance in comparison to moderate CKD (9, 10). However, by CKD III, it is estimated that up to 20% of patients have mild CI and up to 30% of patients with CKD IV have CI when screened with the Mini-Mental Status Exam (MMSE) or Montreal Cognitive Assessment (MoCA) (11, 12). CI is less prevalent in patients with early-stage CKD suggesting that there is a critical window for intervention to prevent progression of both CKD and CI.

CKD patients experience a disproportionately greater number of vascular risk factors than the general population, including T2DM, hypertension, congestive heart failure, and cerebrovascular disease (7, 10, 13, 14). Vascular risk factors are a major contributor to the development of CI (14, 15). Not surprisingly, CI steadily worsens with decline in eGFR (9, 13, 16). This triangulation of CKD, comorbid illnesses that lead to vascular disease, and aging are key contributors to the development and progression of CI. For example, CKD patients are at a 27% risk of atrial fibrillation compared to the general population's 10% risk (14). Atrial fibrillation places CKD patients at higher likelihood of developing cerebrovascular disease, which is a significant factor in CI development. CKD patients also have both an independently increased risk of stroke when eGFR is <60 ml/min/1.73 m^2^ and when macro-albuminuria is present (10, 17, 18). This can be correlated with increased microangiopathy and silent infarcts identified in white matter lesions on MRI in patients with lower eGFR (14).

Albuminuria is another aspect of CKD which has been repeatedly linked to risk of CI (19–22). A prospective cohort study found higher levels of albuminuria to be associated with increased risk of incident dementia in a cohort of 9,967 patients age 54–75 followed for a mean of 18 years independently of dementia risk factors such as hypertension and diabetes (23). Data from the ONTARGET and TRANSCEND study populations were used to assess the relationship between albuminuria and CI, and the effect of ACEi or ARB therapy on albuminuria and CI (19). They included 28,384 participants with vascular disease or diabetes, all of whom underwent MMSE and urine testing for albuminuria at baseline and 5 year follow up. They found that those with micro-albuminuria and macro-albuminuria were at higher risk of having a reduced MMSE score <24 (OR 1.26 95% CI 1.11–1.44 micro-albuminuria, OR 1.49 95% CI 1.20–1.85 macro-albuminuria). Patients with baseline macro-albuminuria who received ACEi or ARB therapy were at a lower risk of MMSE decline compared with patients treated with placebo (19). These findings support the notion that both albuminuria and CI may share common pathogenic factors and CI may respond to therapies that reduce albuminuria such as ACEi, ARB, and potentially SGLT2i (19).

Sodium glucose transport protein 2 inhibitors (SGLT2i) improve cardiovascular outcomes and reduce the effects of vascular risk factors (24–26). RCT evidence demonstrates that SGLT2i prevent diabetes, albuminuria, stroke, and cardiovascular mortality in CKD patients. SGLT2i are a class of medications which increase urinary glucose excretion by inhibiting the sodium-glucose cotransporter 2 (26). They lower blood pressure through by an osmotic effect, decreasing plasma volume and natriuresis. Randomized controlled trials of several SGLT2i showed protective effects including all-cause mortality, decrease in death from cardiovascular disease, hospitalization for heart failure and all-cause mortality (26–28). Studies including DAPA-CKD which showed decreased composite risk of sustained decline in eGFR of at least 50%, ESKD, or death from renal or cardiovascular causes, and DAPA-HF showing decrease in cardiovascular death and worsening heart failure in those with reduced ejection fraction have led to changes in practice and guidelines (24, 29–31). As such, they have become standard of care in patients with type 2 diabetes (T2DM) and cardiovascular disease (32, 33). With the effects these medications have on cardiovascular outcomes, blood pressure, glycemic control, CKD and albuminuria (26, 27, 34), one can speculate that these protective effects could extend to prevention of CI. In this review, we outline the factors associated with CI in CKD and the potential benefits of SGLT2i in the prevention and management of CI in CKD.

## SGLT2i: a Novel Therapy for Vascular Risk Factors and Renoprotection

The SGLT2i include dapagliflozin, canagliflozin, empagliflozin, ertugliflozin, ipragliflozin, luseogliflozin, and tofogliflozin. SGLT2i inhibit the SGLT2 enzyme on the apical surface of segments 1 and 2 of the proximal convoluted tubule of the nephron that reabsorbs glucose back into the blood, inducing glucosuria and natriuresis (35). Potential adverse events related to SGLT2i use include urinary tract infections, genital yeast infections, and euglycemic diabetic ketoacidosis (36–38). Reduction of intravascular volume through diuresis and natriuresis have made them an adjunct to diuretics in hypertension and heart failure (30, 39). The EMPA-REG RCT enrolled over seven thousand patients with T2DM. Patients in the trial were given 10 mg or 25 mg of empagliflozin, or placebo (26). Patients in the empagliflozin group had significantly improved outcomes including improved cardiovascular and all-cause mortality outcomes. Both superiority and non-inferiority analyses were significantly in favor of empagliflozin with respect to death from non-fatal myocardial infarction and non-fatal stroke (26). CANVAS included over nine thousand patients enrolled in the trial with 29% prescribed canagliflozin (40). The participant mean eGFR was 75 ml/min/1.73 m^2^ and mean urine albumin to creatinine ration (ACR) was 12.3 mg/g. Patients in the canagliflozin treatment group had significantly improved outcomes in fatal and cardiovascular outcomes, fatal and non-fatal stroke, progression of albuminuria and use of renal replacement therapy (34). The CREDENCE RCT studied the effect of SGLT2i canagliflozin on over four thousand patients with T2DM and albuminuria with eGFR <30 ml/min/1.73 m^2^ and also treated with renin and angiotensin blockade with primary outcomes of ESKD, doubling of serum creatinine or death from renal or cardiovascular causes after being followed for almost 3 years decreasing mortality by 30% (41).

## SGLT2i Inhibit Pro-inflammatory Pathways in Neurons of Animal Models of Vascular Disease

Recently, the SGLT2 protein has been shown to be implicated in CI-related neuronal pathways in animal models. Several studies demonstrated that SGLT2 inhibition results in amelioration of signaling involved in oxidative stress pathways. It's been hypothesized that the SGLT2 protein has a binding site for acetylcholinesterase in computational biology studies and it's through this mechanism that SGLT2i serve a role in both blood glucose regulation and cognition (42, 43). Lin et al. published one of the first animal studies examining the effects of SGLT2i on cardiovascular, renal, and cognitive outcomes in an obese mouse model (44). Mice treated with empagliflozin for 10 weeks were found to have decreased cardiac and coronary interstitial fibrosis thought to be associated with a reduction in oxidative stress. Lin et al. also examined the cognitive effects of empagliflozin on their mice. Cognition was assessed by the Morris water maze test and was found to be impaired in diabetic mice. Mice given empagliflozin had improved performance after treatment with SGLT2i, suggesting a role for empagliflozin in preservation and/or improvement in cognitive function (44).

Both empagliflozin and dapagliflozin improve cognition in animal models of dementia and CI with a high fat diet. SGLT2 inhibition in rodent studies of neuroprotection in diabetes is significantly more effective than other classes of medications such as dipeptidyl peptidase-4 (DPP4) inhibitors (45). Sa-Nguanmoo et al. used a rat model of high-fat diet induced diabetes to investigate the effects of DPP4 inhibitors vs. SGLT2i on insulin resistance and cognitive function (45). Dapagliflozin administered to rats at a dose of 1 mg/kg showed improved hippocampal synaptic plasticity in comparison to the DPP4 treated group. Furthermore, in rats fed a high fat diet, a marker of inflammation (NFkB) activity, decreased with SGLT2i treatment. Their results demonstrated that SGLT2i were more effective in comparison to the DPP4 inhibitors at improving hippocampal synaptic plasticity in rats fed a high fat diet. The researchers hypothesize the improvement in neuronal plasticity occurred through prevention of insulin resistance and decreased neuronal apoptosis in the SGLT2i group (45).

Aside from reducing inflammation, SGLT2i may reduce CI through their role in energy metabolism pathways such as the mTOR pathway. The mTOR pathway is linked to changes in the regulation of anabolism and catabolism, especially nocturnal regulation of homeostasis of glycemic pathways (46). The SGLT2i role in regulation of the mTOR pathway is in its glucosuria, which is hypothesized to confer more favorable outcomes for mTOR signaling that could relate to a decrease in CI. The theory from Esterline et al. regarding SGLT2i improvements in multi-organ function relate to its glucosuria that is hypothesized to restore diurnal switching between anabolic and catabolic states by mTOR signaling (46). The increase in glycogenolysis and gluconeogenesis with SGLT2i decreases mTOR signaling (46).

Lastly, SGLT2 may reduce the physical disruption of neurons. In a murine model of AD and T2DM, researchers demonstrated treatment with empagliflozin at a dose of 10 mg/kg for 22 weeks reduced neuronal loss on necropsy (47). Specifically, SGLT2 treated mice showed reductions in amyloid plaques and tau protein (47). The investigators hypothesized that a decrease in cortical thinning is due to improved glycemic control with empagliflozin. In addition to histopathological differences, empagliflozin-treated mice also demonstrated significant improvement in their memory and learning performance.

## SGLT2i May Reduce CI Through Reduction in Cerebrovascular Disease

Macrovascular complications of diabetes such as stroke play a significant role in the development of CI (27, 40). From the major RCTs for SGLT2i and cardiovascular outcomes, it was previously thought that SGLT2i had a neutral effect on stroke outcomes (48). In a meta-analysis of the RCTs of SGLT2i, Tsai et al. performed a subgroup analysis of stroke outcomes (48). Their search included 5 studies including EMPA-REG OUTCOME, CANVAS, DECLARE TIMI 58, and VERTIS where number of participants ranged from 4,000 to 17,000 from 2 to 4 years study length (48). SGLT2i were not associated with a reduction in ischemic stroke, transient ischemic attack, or fatal stroke, but that use of SGLT2i may be associated with a significant 50% reduction in hemorrhagic stroke (RR = 0.49, 95% CI 0.30–0.82, *P* = 0.007); however, this was among a small absolute number of events (48).

CKD and micro-albuminuria increase patients' risk of hemorrhagic and ischemic stroke. In a meta-analysis of the CREDENCE trial, subgroups with diabetes and atrial fibrillation were assessed for stroke events using a *post-hoc* analysis (49). There was a total of 142 patients diagnosed with stroke during the trial (49). It was determined that patients with decreased eGFR influenced SGLT2i effects on stroke. For patients included in the analysis with the lowest renal function (eGFR <45 ml/min/1.73 m^2^), there was evidence for protective effect in the SGLT2i treatment group (49). They concluded from their subgroup analysis that there may be benefit for protection against hemorrhagic stroke prevention for patients with CKD.

A study reporting a significant association between cognitive impairment and short term SGLT2i use was recently published. A single center RCT from Mone et al. examined the effects of SGLT2i on frail elderly with diabetes and heart failure with preserved ejection fraction (HFpEF) (50). In their study, seniors were randomized to empagliflozin (N = 52), metformin, or insulin and administered the MoCA at baseline and 1 month after treatment (50). The study included adults >65 with MoCA scores <26 and approximately one third of their participants had previous diagnoses of CKD (50). There was a significant improvement in MoCA sores in the empagliflozin treatment group: The mean MoCA scores in the three groups at baseline and 1-month follow-up were 19.80 ± 3.77 vs. 22.25 ± 3.27 (*P* < 0.001) in the empagliflozin group (50). The metformin and insulin groups did not have significant improvement in MoCA scores. This study provides promising evidence that empagliflozin may have CI benefits over other diabetes treatments (50).

## Discussion

CI is common in patients with CKD and CI may be preventable by targeting vascular risk factors associated with CKD progression. A relatively new treatment for CKD and cardiovascular disease has become available, allowing Nephrologists to potentially target multiple vascular risk factors for CI in CKD with one medication. SGLT2i demonstrate significant benefit in reducing all-cause mortality and cardiovascular adverse outcomes in virtually all published SGLT2i RCTs.

However, the understanding of SGLT2i's putative role in cognitive impairment is in its very early stages. In animal model studies published to date, SGLT2i-treated rodents have improved memory performance. There are few studies of animal model cognitive function after treatment with SGLT2i, however, these studies suggest that use of SGLT2i are protective from CI mainly through regulation of blood glucose and decreased activity of inflammatory pathways. Why do the human participant RCTs and the animal model evidence differ with respect to their results regarding SGLT2i putative involvement in preventing cognitive impairment? Animal models showed potential for SGLT2i involvement in cognition whereas the RCTs showed some significant composite all-cause mortality results when non-fatal stroke was included. There were no CI outcomes included in the major human SGLT2i RCTs. There may be several reasons to account for this discrepancy: The animal model studies had shorter study durations and larger doses of the SGLT2i (i.e., 1 mg/kg) than the human RCTs. The N number of animal models is much smaller than RCTs with possibility that the neurological improvements in the animal studies may bias the results due to large variability and low reliability.

It is difficult to compare or conclude that CI can be improved from the limited RCT data we have at this time. We could hypothesize that SGLT2i function to improve CI risk through their reduction in vascular risk factors based on the outcomes of the trials. For example, CREDENCE primary endpoints were cardiovascular and renal disease and included stroke as a secondary outcome without inclusion of CI, dementia as secondary outcomes (25). Similarly, both EMPA-REG-OUTCOME and CANVAS did not examine outcomes in cognitive impairment but did have a significant primary outcome of non-fatal stroke (26, 27). In DAPA-CKD, patients with stroke or TIA were excluded from the study and CI, CVA or related adverse event was not included in their study analysis; however, significant reduction in renal and cardiovascular mortality demonstrated suggests dapagliflozin improves vascular risk factors (24). We do not know if SGLT2i directly impact CI development in patients with CKD, but the RCT data show compelling data to choose SGLT2i in patients with CI/vascular risk factors.

We now have the first preliminary evidence to suggest that SGLT2i improve CI in patients with T2DM (50). SGLT2i may be associated with neuroprotection including possible reduction in inflammation, and inhibition of acetylcholinesterase (50). To this end, there is increasing interest in further examination of SGLT2i role in CI. The EMPA REG ELDERLY is underway in Japan using the MMSE-J to measure CI and may provide further support for the use of SGLT2i in the elderly for CI protection (51). Results of these studies will provide further insight into the promising involvement of SGLT2i in prevention of CI.

CI is a progressive, and irreversible disease that is prevalent in older patients with CKD. Vascular risk factors for CI including hypertension, diabetes, cardiovascular disease, albuminuria, and cerebrovascular disease can be preventable if early intervention occurs. SGLT2i are an emerging therapy to manage and prevent all of these risk factors. Like ACEi and ARBs, SGLT2i decrease albuminuria, which may have benefit in prevention of CI. SGLT2i demonstrate compelling evidence for reduction in mortality and adverse cardiovascular outcomes in RCTs and a modest, but significant reduction in hemorrhagic stroke in RCT sub-analysis. Clinicians could consider earlier use of SGLT2i in older patients to provide cardiovascular and renoprotective benefits with potential downstream cognition benefits. Further longitudinal data are needed to determine if SGLT2i have a similarly protective effect against CI in CKD.

## Author Contributions

JN was responsible for drafting of the initial manuscript. IH contributed to the initial draft. MS was the supervisor. All authors contributed to the critical revision of the manuscript.

## Conflict of Interest

The authors declare that the research was conducted in the absence of any commercial or financial relationships that could be construed as a potential conflict of interest.

## Publisher's Note

All claims expressed in this article are solely those of the authors and do not necessarily represent those of their affiliated organizations, or those of the publisher, the editors and the reviewers. Any product that may be evaluated in this article, or claim that may be made by its manufacturer, is not guaranteed or endorsed by the publisher.

## Abbreviations

AD: Alzheimer's dementia
ACEi: Angiotensin converting enzyme inhibitor
ACR: Albumin to creatinine ratio
ARB: Angiotensin II receptor blocker
CI: Cognitive impairment
CKD: Chronic kidney disease
DPP4: Dipeptidyl peptidase-4
eGFR: Estimated glomerular filtration rate
ESKD: End stage kidney disease
HFpEF: Heart failure with preserved ejection fraction
MMSE: Mini-mental status exam
MoCA: Montreal Cognitive Assessment
RCT: Randomized controlled trial
SGLT2i: Sodium glucose transporter 2 inhibitor
T2DM: Type 2 diabetes mellitus.
